# Supplementary material for: Medical Admission Prediction Score (MAPS); a simple tool to predict medical admissions in the emergency department
Source: PLoS One. 2023 Nov 10;18(11):e0293140. doi: 10.1371/journal.pone.0293140 (PMC10637671; doi:10.1371/journal.pone.0293140)
Supplement: S1 Table — (DOCX) [file pone.0293140.s001.docx]

Supplementary Table 1: Details of the Area under the curve

| **Area Under the Curve** | | | | |
| --- | --- | --- | --- | --- |
| Test Result Variable(s): | | | | |
| Area | Std. Error^a^ | Asymptotic Sig.^b^ | Asymptotic 95% Confidence Interval | |
|  |  |  | Lower Bound | Upper Bound |
| **0.831** | 0.002 | 0.000 | **0.827** | **0.836** |
| The test result variable(s): Total weighted scores has at least one tie between the positive actual state group and the negative actual state group. Statistics may be biased. | | | | |
| a. Under the nonparametric assumption | | | | |
| b. Null hypothesis: true area = 0.5 | | | | |
|  |  |  |  |  |
|  |  |  |  |  |
| **Coordinates of the Curve** | | |  |  |
| Test Result Variable(s): | | |  |  |
| Positive if Greater Than or Equal To^a^ | Sensitivity | 1 - Specificity |  |  |
| 2.00 | 1.000 | 1.000 |  |  |
| 3.50 | 1.000 | 1.000 |  |  |
| 4.50 | 1.000 | 0.996 |  |  |
| 5.50 | 1.000 | 0.995 |  |  |
| 6.50 | 1.000 | 0.994 |  |  |
| 7.50 | 1.000 | 0.991 |  |  |
| 8.50 | 1.000 | 0.947 |  |  |
| 9.50 | 0.854 | 0.366 |  |  |
| 10.50 | 0.852 | 0.359 |  |  |
| 11.50 | 0.852 | 0.357 |  |  |
| 12.50 | 0.852 | 0.355 |  |  |
| 13.50 | 0.847 | 0.343 |  |  |
| 14.50 | 0.819 | 0.308 |  |  |
| 15.50 | 0.800 | 0.295 |  |  |
| 16.50 | 0.797 | 0.283 |  |  |
| **17.50** | **0.691** | **0.162** |  |  |
| 18.50 | 0.645 | 0.133 |  |  |
| 19.50 | 0.644 | 0.132 |  |  |
| 20.50 | 0.642 | 0.129 |  |  |
| 21.50 | 0.608 | 0.113 |  |  |
| 22.50 | 0.527 | 0.075 |  |  |
| 23.50 | 0.484 | 0.063 |  |  |
| 24.50 | 0.469 | 0.059 |  |  |
| 25.50 | 0.463 | 0.055 |  |  |
| 26.50 | 0.386 | 0.038 |  |  |
| 27.50 | 0.359 | 0.031 |  |  |
| 28.50 | 0.310 | 0.022 |  |  |
| 29.50 | 0.299 | 0.020 |  |  |
| 30.50 | 0.280 | 0.016 |  |  |
| 31.50 | 0.258 | 0.014 |  |  |
| 32.50 | 0.222 | 0.010 |  |  |
| 33.50 | 0.207 | 0.009 |  |  |
| 34.50 | 0.188 | 0.007 |  |  |
| 35.50 | 0.169 | 0.005 |  |  |
| 36.50 | 0.155 | 0.004 |  |  |
| 37.50 | 0.139 | 0.003 |  |  |
| 38.50 | 0.134 | 0.003 |  |  |
| 39.50 | 0.125 | 0.003 |  |  |
| 40.50 | 0.113 | 0.002 |  |  |
| 41.50 | 0.101 | 0.002 |  |  |
| 42.50 | 0.091 | 0.001 |  |  |
| 43.50 | 0.087 | 0.001 |  |  |
| 44.50 | 0.079 | 0.001 |  |  |
| 45.50 | 0.074 | 0.001 |  |  |
| 46.50 | 0.063 | 0.001 |  |  |
| 47.50 | 0.058 | 0.001 |  |  |
| 48.50 | 0.051 | 0.000 |  |  |
| 49.50 | 0.047 | 0.000 |  |  |
| 50.50 | 0.042 | 0.000 |  |  |
| 51.50 | 0.037 | 0.000 |  |  |
| 52.50 | 0.034 | 0.000 |  |  |
| 53.50 | 0.030 | 0.000 |  |  |
| 54.50 | 0.028 | 0.000 |  |  |
| 55.50 | 0.024 | 0.000 |  |  |
| 56.50 | 0.021 | 0.000 |  |  |
| 57.50 | 0.019 | 0.000 |  |  |
| 58.50 | 0.017 | 0.000 |  |  |
| 59.50 | 0.015 | 0.000 |  |  |
| 60.50 | 0.013 | 0.000 |  |  |
| 61.50 | 0.012 | 0.000 |  |  |
| 62.50 | 0.010 | 0.000 |  |  |
| 63.50 | 0.009 | 0.000 |  |  |
| 64.50 | 0.007 | 0.000 |  |  |
| 65.50 | 0.006 | 0.000 |  |  |
| 66.50 | 0.005 | 0.000 |  |  |
| 67.50 | 0.004 | 0.000 |  |  |
| 68.50 | 0.004 | 0.000 |  |  |
| 69.50 | 0.003 | 0.000 |  |  |
| 70.50 | 0.003 | 0.000 |  |  |
| 71.50 | 0.003 | 0.000 |  |  |
| 72.50 | 0.002 | 0.000 |  |  |
| 73.50 | 0.002 | 0.000 |  |  |
| 75.00 | 0.001 | 0.000 |  |  |
| 76.50 | 0.001 | 0.000 |  |  |
| 77.50 | 0.001 | 0.000 |  |  |
| 78.50 | 0.001 | 0.000 |  |  |
| 79.50 | 0.001 | 0.000 |  |  |
| 80.50 | 0.001 | 0.000 |  |  |
| 82.00 | 0.000 | 0.000 |  |  |
| 84.00 | 0.000 | 0.000 |  |  |
| 85.50 | 0.000 | 0.000 |  |  |
| 87.00 | 0.000 | 0.000 |  |  |
| The test result variable(s): Total weighted scores has at least one tie between the positive actual state group and the negative actual state group. | | |  |  |
| a. The smallest cutoff value is the minimum observed test value minus 1, and the largest cutoff value is the maximum observed test value plus 1. All the other cutoff values are the averages of two consecutive ordered observed test values. | | |  |  |
